# Sugammadex Vs. Neostigmine for Cerebral Perfusion During Emergence From General Anesthesia in Patients Undergoing Carotid Endarterectomy: A Double‐Blind Randomized Controlled Trial

**DOI:** 10.1002/cns.70924

**Published:** 2026-05-18

**Authors:** Xuefei Jia, Wenbo Cao, Tianying Li, Qinghai Liu, Yi An, Lixia Li, Zhongjia Li, Chuanyu Liang, Pei Wang, Hongyi Song, Ke Cui, Manke Luo, Shuning Zhang, Xiaoxu Chen, Zhiwei Wu, Boyuan Li, Tianlong Wang, Liqun Jiao, Xiaoyang Li, Lei Zhao

**Affiliations:** ^1^ Department of Anesthesiology Xuanwu Hospital, Capital Medical University Beijing China; ^2^ The Second School of Clinical Medicine Southern Medical University Guangzhou China; ^3^ The Affiliated Guangdong Second Provincial General Hospital of Jinan University Guangzhou China; ^4^ School of Reliability and Systems Engineering Beihang University Beijing China; ^5^ Science and Technology on Reliability and Environmental Engineering Laboratory Beijing China; ^6^ Department of Neurosurgery Xuanwu Hospital, Capital Medical University, No. 45 Changchun Street, Xicheng District Beijing China

**Keywords:** anesthesia emergence, carotid endarterectomy, cerebral hyperperfusion, neostigmine, neuromuscular blockade, sugammadex

## Abstract

**Background:**

Carotid endarterectomy (CEA) is the standard treatment for carotid stenosis, but has the risk of cerebral hyperperfusion syndrome (CHS). Postoperative residual neuromuscular blockade can trigger cerebral hyperperfusion (CH), and the effects of neostigmine and sugammadex on cerebral perfusion during CEA are unclear.

**Objective:**

This study aimed to compare the efficacy of neostigmine combined with atropine and sugammadex in reversing neuromuscular blockade in CEA patients under general anesthesia and evaluate the superiority of sugammadex in maintaining normal cerebral perfusion during anesthesia emergence.

**Methods:**

A single‐center, prospective, randomized controlled study was carried out and registered on the Chinese Clinical Trial Registry website (ChiCTR2300078579). Between December 2023 and June 2024, 90 patients were recruited. After exclusions, 82 patients were randomly assigned to the sugammadex group or the neostigmine group at a 1:1 ratio. The primary outcome was the difference in the area under the curve of the percentage of middle cerebral artery velocity relative to baseline (MCAV% S) during emergence. Secondary outcomes included multiple hemodynamic and clinical parameters, as well as pulmonary and CH‐related complications within 48 h post‐operation. Mediation analysis, ROC analysis, and multivariate linear regression analysis were used to illustrate the differences in MCAV%S between the two groups and subsequent analysis.

**Results:**

The MCAV% S in the sugammadex group was significantly lower than in the neostigmine group (24,600 [14,940–38,040] vs. 31,575 [24,210–50,040], *p* = 0.044), and this difference persisted after adjusting for variables (Estimate (95% CI): −10,896 [−21,387 ~ −405]; *p* = 0.042). The sugammadex group had shorter eye‐opening and extubation times, and a lower incidence of hypoxemia and overall pulmonary complication score. Mediation analysis showed that extubation time mediated part of the effect on MCAV% S, and the ROC analysis indicated good predictive performance of MCAV% S for CH.

**Conclusion:**

In CEA patients, sugammadex is more advantageous than neostigmine in reversing neuromuscular blockade during emergence, as it shortens extubation time, reduces excessive cerebral blood flow, and potentially lowers the incidence of postoperative complications.

## Graphical abstract

In this double‐blind RCT involving 82 patients undergoing carotid endarterectomy, sugammadex significantly reduced excessive cerebral blood flow during emergence from general anesthesia compared to neostigmine (MCAV% S: 24,600 vs. 31,575, *p* = 0.044). Sugammadex also shortened extubation time (110 vs. 237 s, *p* < 0.001) and lowered the incidence of postoperative hypoxemia (12.2% vs. 31.7%, *p* = 0.033). Mediation analysis confirmed that shorter extubation time partially mediated the cerebral protective effect of sugammadex.

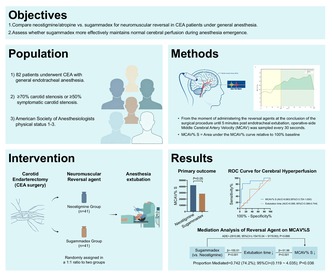

## 1 Introduction

Stroke imposes a significant burden on global health, ranking as the second leading cause of death. Approximately 87% of these cases are ischemic strokes, which may result in severe disability or death [1, 2]. Carotid endarterectomy (CEA) is the predominant procedure for managing ischemic stroke and is recognized as the gold standard treatment for asymptomatic and symptomatic carotid stenosis [2, 3]. Cerebral hyperperfusion (CH) occurs postoperatively when cerebral blood flow increases by more than 100% compared to baseline, often progressing to cerebral hyperperfusion syndrome (CHS) [4]. CHS is characterized by a distinct triad of migraine, focal neurological deficit, and seizures without cerebral ischemia following CEA [5]. This condition represents a severe complication of CEA; about 40% of affected patients may experience cerebral hemorrhage or death if not promptly detected and managed [6]. Currently, no definitive treatment guidelines exist for CHS, and the efficacy of available treatments remains uncertain [7], so prevention is paramount.

Postoperative residual neuromuscular blockade is considered a potential cause of CH following CEA. This condition refers to the incomplete recovery of muscle strength due to the presence of neuromuscular blocking agents or their metabolites. Residual neuromuscular blockade can lead to respiratory muscle weakness, CO_2_ accumulation, increased cerebral blood flow and velocity, thereby leading to CH. Neostigmine, a cholinesterase inhibitor, is commonly used to reverse neuromuscular block. However, it carries unavoidable side effects such as bradycardia and cholinergic crisis, and is ineffective against deep neuromuscular block. Sugammadex, a synthetic modified γ‐cyclodextrin and a specific reversal agent for rocuronium, has been shown to reverse neuromuscular blockade more effectively and rapidly, decrease time to extubation [8], and reduce postoperative residual neuromuscular blockade, potentially decreasing the incidence of CH following CEA. Additionally, sugammadex can reverse deep neuromuscular blockade [9, 10]. Research involving sugammadex has been conducted across various surgeries, including laparoscopic, breast, and spinal surgeries, demonstrating reduced postoperative pulmonary complications and shortened hospital stays [11, 12, 13, 14]. However, related research specific to CEA remains scarce.

Therefore, this study aims to compare the efficacy of neostigmine combined with atropine vs. sugammadex in reversing neuromuscular blockade in patients undergoing CEA under general anesthesia, and to determine whether sugammadex is superior to neostigmine in maintaining normal cerebral perfusion during anesthesia emergence.

## 2 Methods

This is a single‐center, prospective, randomized controlled study. Ethical approval for this study (No. [2023]140) was provided by the Ethics Committee of Xuanwu Hospital, Capital Medical University. Patient recruitment took place from December 2023 to June 2024. Before recruitment, the trial was registered via the Chinese Clinical Trial Registry website (trial registration code: ChiCTR2300078579). There were no protocol changes after trial commencement. Written informed consent was obtained before patient enrolment. The findings and procedures of this study have been reported in strict accordance with the Consolidated Standards of Reporting Trials (CONSORT 2010) Guidelines [15].

### 2.1 Patients

**FIGURE 1:**
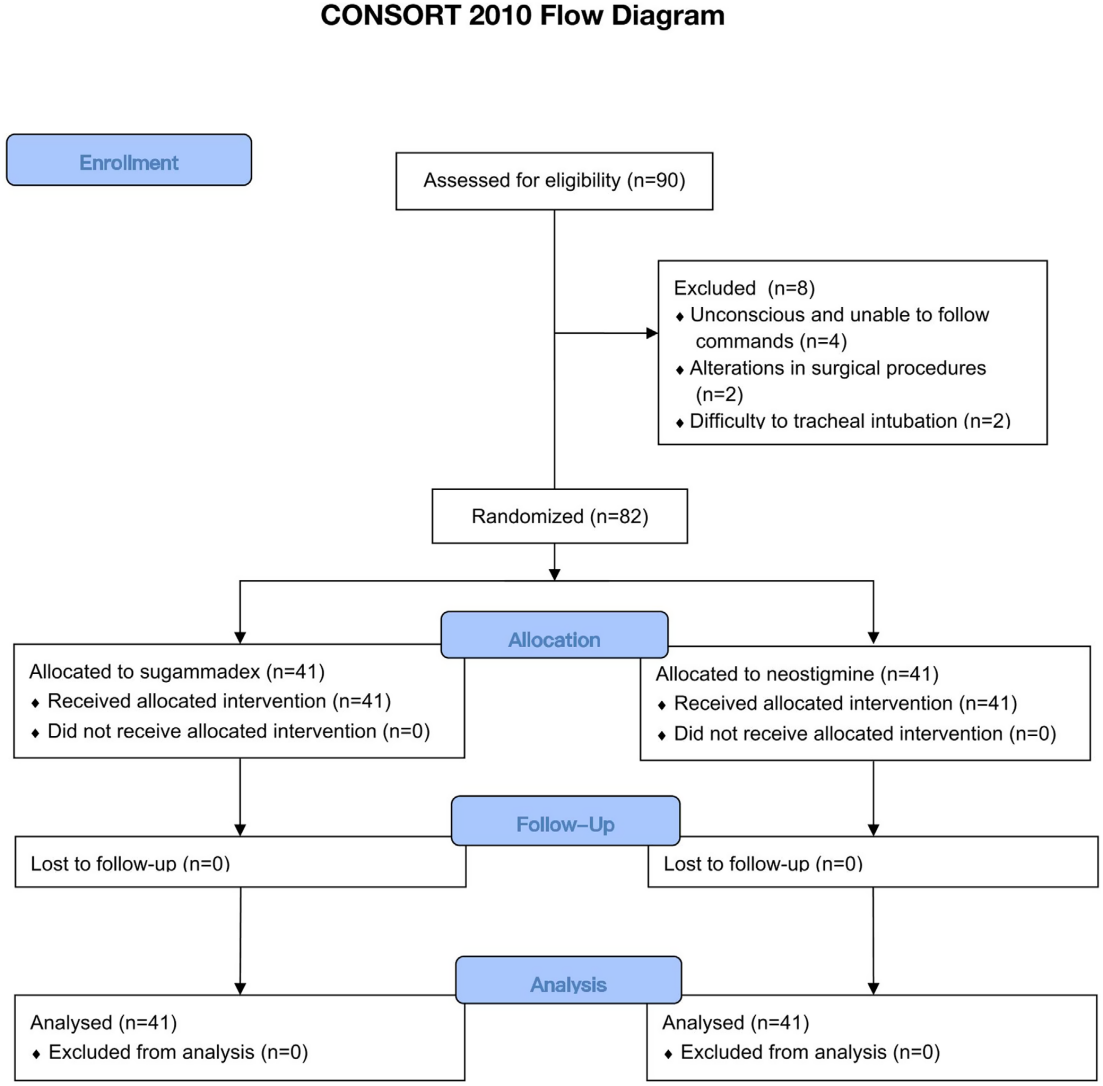
Consolidated Standards of Reporting Trials flow diagram.

We included patients with carotid artery stenosis reaching 70% or symptomatic carotid artery stenosis of 50% [16]; who underwent CEA under general anesthesia with endotracheal intubation and American Society of Anesthesiologists (ASA) physical status of 1 to 3. The exclusion criteria were as follows: Patients with severe cardiac, pulmonary, hepatic, or renal dysfunction (Detailed definitions of severe organ dysfunction are provided in the Supporting Information); those suffering from neuromuscular disorders caused by various factors; individuals with allergies to rocuronium, neostigmine, or sugammadex; patients experiencing critical intraoperative conditions such as hemorrhage and malignant arrhythmias; those who were unconscious and unable to follow commands; patients with a suspected difficult airway; cases where there were alterations in surgical procedures (including intraoperative shunting, postoperative carotid artery stenting, or angiography during surgery); those who had participated in other studies or refused to participate in this study, and patients who underwent concurrent surgeries. The patient screening flowchart is presented as Figure 1 in this study.

### 2.2 Randomization and Masking

Participants were randomized into the sugammadex group and the neostigmine group in a 1:1 ratio. The randomization process was carried out using the random number table method by an anesthesiologist who was not involved in either the anesthesia management or postoperative follow‐up. Before extubation, an appropriate dose of the assigned reversal agent, determined based on the patient's weight, was diluted to 10 mL with normal saline and loaded into an unlabeled 10 mL syringe. The anesthesiology team administered the pre‐drawn reversal agent after the surgery. In this study, all anesthetic procedures were performed by anesthesiologists from the same team, and the patients were kept uninformed about the specific drugs used. Data recording and patient follow‐up were conducted by anesthesiologists who were unaware of the group assignments and not engaged in the anesthesia management. The ultrasound doctors who performed the TCD examination were not involved in the grouping assignment, were unaware of the group assignments, and were not involved in anesthesia management. Ultrasound data were reported by experts who were not affiliated with the research team. Subsequent data processing was performed by independent professionals who were not involved in the measurements and were completely blinded to group allocation and clinical data.

### 2.3 Procedure

Patients underwent monitoring that included electrocardiography, pulse oximetry, non‐invasive and invasive blood pressure (IBP), and BIS monitoring at admission. Anesthesia was induced using propofol, sufentanil, and rocuronium. Neuromuscular blockade was monitored throughout the operation using the Philips MX700 neuromuscular blockade monitoring module (865,383 Philips, Shanghai, China). Before anesthesia induction, the patient's forearm was secured, and the skin was degreased. Adductor pollicis muscle with electrodes placed over the ulnar nerve at the wrist; the negative electrode near the wrist, and the positive electrode 2~3 cm centripetally. A palm adapter connected the sensor and thermometer to the patient's palm, which remained immobile during monitoring. If signal instability (e.g., waveform loss or unresponsive stimulation) was observed, the electrodes were checked and repositioned if necessary; the sensor was also reattached to ensure proper thumb movement. Following propofol administration, baseline TOFr was recorded; subsequently, 0.6 mg/kg rocuronium was administered, and endotracheal intubation was performed at TOFc ≤ 1. Then, ultrasound physicians calibrated the mean velocity of the middle cerebral artery (MCAV) using an EMS‐9 PB ultrasound monitor (Delica, Shenzhen, China; software version V1.0), while anesthesiologists calibrated IBP and heart rate (HR). Rocuronium was administered in a dose of 0.2 mg/kg when the train‐of‐four ratio (TOFr) ≥ 0.2 and was not administered if less than 30 min remained in the surgery. Throughout the operation, BIS levels were maintained between 40 and 60, and PaCO_2_ between 35 and 45 mmHg. At the end of the procedure, upon achieving TOFc ≥ 2, the experimental group received sugammadex at 2 mg/kg, while the control group received neostigmine at 0.02 mg/kg combined with atropine at 0.01 mg/kg. Extubation was considered when TOFr reached ≥ 0.9 [10]. Patients were then transferred to the post‐anesthesia care unit (PACU) for continued monitoring. (Details in Supplementary).

### 2.4 Data Collection

Baseline characteristics, perioperative laboratory examinations, medication administration, and intraoperative variables were prospectively collected for all study participants.

General demographic and clinical data included age, sex, operative side, symptomatic stenosis, and preoperative modified Rankin Scale (mRS) score. The severity of stenosis on the surgical side and the non‐operative side was documented, together with collateral circulation status on the operative side.

Comorbidities were recorded, including diabetes, hypertension, history of cerebral infarction, cardiac disease, and dyslipidemia. Lifestyle risk factors consisted of smoking and drinking status. Preoperative medications were collected in detail, comprising antihypertensive drugs, oral hypoglycemic agents, insulin, antiplatelet agents, anticoagulants, and statins.

Preoperative laboratory assessments included hemoglobin, erythrocyte count, white blood cell count, platelet count, urea nitrogen, serum creatinine, SGOT, SGPT, fasting blood glucose, serum sodium, potassium, and chloride levels.

Intraoperative data covered the dosages of anesthetic agents, including propofol, sufentanil, remifentanil, dexmedetomidine, and rocuronium. Intraoperative fluid management parameters included crystalloid solution, colloidal solution, blood loss, urine volume, and total infusion volume. Anesthesia duration, operation duration, and vascular clamp time were also recorded.

Additional perioperative indicators included ASA physical status, Charlson score, patient position during the peri‐extubation period, PetCO2 changes, and TOFr calibration value.

**FIGURE 2:**
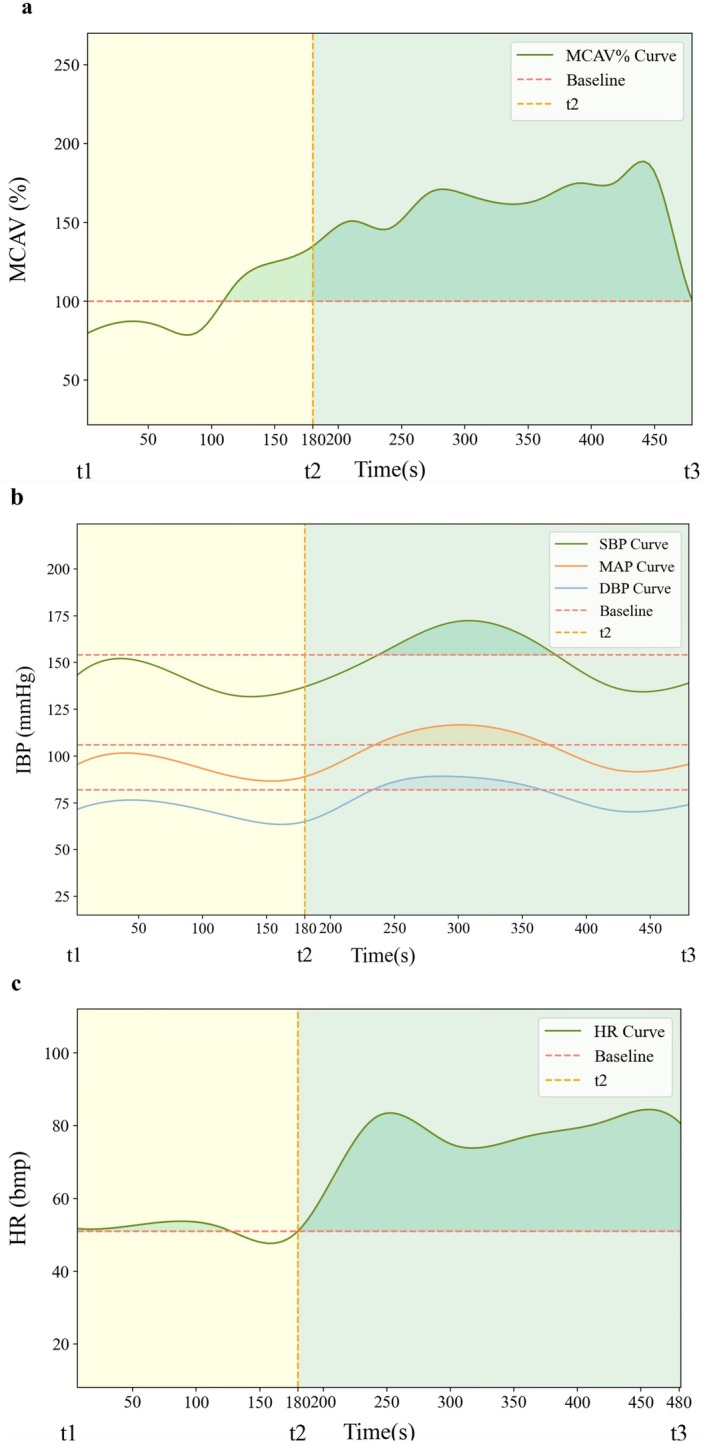
Area under the curve (AUC) for MCAV% during peri‐extubation period in one representative patient (a); AUC for IBP in one patient during peri‐extubation period (b); AUC for HR in one patient during peri‐extubation period (c); Line chart of the mean values of two drugs over time (d). t1: Reversal agents administered at the end of surgery; t2: At extubation; t3: 5 min post‐extubation.

From the moment of administering the reversal agents at the conclusion of the surgical procedure until 5 min post endotracheal extubation, the middle cerebral artery velocity (MCAV) on the operative side was sampled at 30‐s intervals. All the MCAV% values were then connected to form a smooth curve on the coordinate axis. The area enclosed by this curve and the baseline value (100%) was calculated and recorded as “MCAV% S” (as depicted in Figure 2).

The areas under the curves (calculated using the same method as for MCAV%, but with sampling occurring at 60‐s intervals) for systolic blood pressure (SBP), diastolic blood pressure (DBP), mean arterial pressure (MAP), and heart rate (HR) were designated as “SBP S”, “DBP S”, “MAP S”, and “HR S” respectively. The ratio of PetCO_2_ at 5 min post‐extubation to PetCO_2_ at the time of reversal agent administration was designated as “PetCO_2_ changes”.

### 2.5 Primary and Secondary Outcomes

Diagnostic criteria for CH: CH is defined as a postoperative cerebral blood perfusion exceeding 200% of the baseline [4]. In this study, perioperative cerebral perfusion was monitored by TCD, with evaluations performed by neurosurgeons and vascular ultrasound specialists post‐CEA [17].

Primary outcomes were the difference in MCAV% S between the groups during emergence. Secondary outcomes included differences in area under the curve (AUC) above baseline for IBP, HR (SBP S; DBP S; MAP S; HR S), eye‐opening time, time to extubation, PACU length of stay, end‐tidal carbon dioxide partial pressure (PetCO_2_), and blood gas analysis during the peri‐extubation period. Pulmonary complications (pneumonia, aspiration pneumonia, atelectasis, pneumothorax, hypoxemia, upper respiratory tract obstruction, acute respiratory distress syndrome) [18, 19, 20] were monitored for 48 h post‐operation. CH‐related symptoms (dizziness, headache, hemispheric hemorrhage on the operative side, craniofacial redness and swelling, nausea and vomiting, eye symptoms, convulsions, and transient neurological dysfunction) [4, 21] were monitored for 48 h post‐operation. (Details in Supplementary).

### 2.6 Statistical Analysis

In the pilot study, the mean MCAV% S and standard deviation were 33,391 s · % and 13,554 s · % in the sugammadex group, and 47,897 s · % and 30,973 s · % in the neostigmine group. Setting a significance level at α = 0.05 and a power of 1‐β = 0.8, and accounting for a 10% loss to follow‐up, the sample size was calculated to be 82 (41 for each group). R statistical software, Version 4.4.2, was utilized for data analysis. The Kolmogorov–Smirnov test assessed the normality of all measurement data, and the results are presented in Table S3. Data conforming to normal distribution were analyzed using the *t*‐test and presented as mean and standard deviation, while non‐normal data were evaluated using nonparametric tests and presented as medians with interquartile ranges. A Chi‐square test or Fisher's exact test was applied to assess differences between Categorical variables.

We employed Python 3.11.5 software to calculate the area above the baseline MCAV% through numerical integration using the interpolation method. In the univariate linear regression analysis, we investigated the associations between MCAV% S and groups, extubation time, and PetCO_2_ changes. This analysis aimed to assess the individual effects of each independent variable on the dependent variable. For the multivariate linear regression analysis, covariates were selected based on clinical relevance, and those with a *p*‐value < 0.05 in univariate analysis; variables with clinical relevance were pre‐specified, while variables identified by univariate analysis were added post hoc. We incorporated clinically significant variables or those with *p*‐values < 0.05 from the univariate linear regression into the model. These variables included sex, degree of stenosis on the operative side, collateral of the operative side, anesthesia time, MAP S, HR S, vascular clamp time, and the total volume of intraoperative infusions. Moreover, we performed a mediation analysis to identify the mediating role of potential mediators in the relationship between the independent and dependent variables. Furthermore, we conducted a Receiver Operating Characteristic (ROC) analysis to evaluate the diagnostic performance of the variables under investigation. A *p*‐value < 0.05 was considered statistically significant.

## 3 Results

The baseline characteristics of the patients were presented in Table 1 (The details of participant characteristics were presented in the Table S1). 90 patients were continuously recruited; 4 patients were unable to follow instructions due to unconsciousness, 2 were excluded due to alterations in surgical procedures, and 2 due to a history of head and neck radiotherapy that led to difficulty in tracheal intubation. 82 patients were enrolled in the final analysis and divided into the neostigmine group (*n* = 41) and the sugammadex group (*n* = 41). The flow chart is shown in Figure 1. The mean age (64 ± 8 vs. 64 ± 9 years old, *p* = 0.699) and proportion of females (9.80% vs. 17.10%, *p* = 0.331) had no significant difference between the two groups. The total volume of intraoperative infusions was lower in the sugammadex group compared to the neostigmine group (1200 [1,100, 1300] vs. 1300 [1200, 1600], *p* = 0.038); no significant differences were noted in other baseline characteristics.

**TABLE 1:** Characteristics of included patients.

	Sugammadex (*n* = 41)	Neostigmine (*n* = 41)	*p*
Age (yr), mean ± SD	64 ± 8	64 ± 9	0.699
Female, *n* (%)	4 (9.80)	7 (17.10)	0.331
Symptomatic stenosis, *n* (%)	36 (87.80)	33 (80.50)	0.364
Preoperative mRS Score, *n* (%)			0.319
0–2	28 (68.30)	32 (78.00)	
≥ 3	13 (31.70)	9 (22.00)	
Degree of stenosis on the operative side, *n* (%)			0.775
50%–70%	8 (19.50)	7 (17.10)	
70%–99%	33 (80.50)	34 (82.90)	
Degree of stenosis on the non‐operative side, *n* (%)			0.965
No obvious stenosis	13 (31.70)	14 (34.10)	
May	17 (41.50)	15 (36.60)	
50%–70%	7 (17.10)	7 (17.10)	
70%–99%	4 (9.80)	5 (12.20)	
Collateral of the operative side, *n* (%)			0.676
None	3 (7.5)	1 (2.5)	
Anterior communicating artery	13 (32.5)	16 (40)	
Posterior communicating artery	4 (10)	5 (12.5)	
Both	20 (50)	18 (45)	
Diabetes, *n* (%)	16 (39.00)	14 (34.10)	0.647
Hypertension, *n* (%)	31 (75.60)	27 (65.90)	0.332
History of cerebral infarction, *n* (%)	20 (48.80)	17 (41.50)	0.506
ASA physical status, *n* (%)			0.672
2	4 (9.80)	2 (4.90)	
3	37 (90.20)	39 (95.10)	
Charlson score, median (IQR)	3 (3, 5)	3 (3, 5)	0.683
Rocuronium dosage (mg), mean ± SD	57.51 ± 8.68	57.88 ± 9.28	0.854
Anesthesia time (min), mean ± SD	198.34 ± 32.94	202.34 ± 32.37	0.581
Operation time (min), mean ± SD	138.93 ± 31.04	146.20 ± 32.32	0.302
Vascular clamp time (min), median (IQR)	32 (28, 39)	35 (30, 31)	0.215
Total volume of intraoperative infusions (ml), median (IQR)	1200 (1100, 1300)	1300 (1200, 1600)	0.038
Patient's position during peri‐extubation period, *n* (%)			0.114
Horizontal supine position	35 (85.40)	40 (97.60)	
Head‐down position	6 (14.60)	1 (2.40)	
PetCO_2_ changes	1.11 ± 0.06	1.11 ± 0.07	0.93
TOFr calibration value, mean ± SD	109 ± 12	109 ± 7	0.973

Abbreviations: ASA, American Society of Anesthesiologists; IQR, inter‐quartile range; SD, standard deviation; TOFr, train‐of‐four ratio.

All data were carefully documented and validated to guarantee reliability for further statistical analysis.

### 3.1 Primary and Secondary Outcomes

**FIGURE 3:**
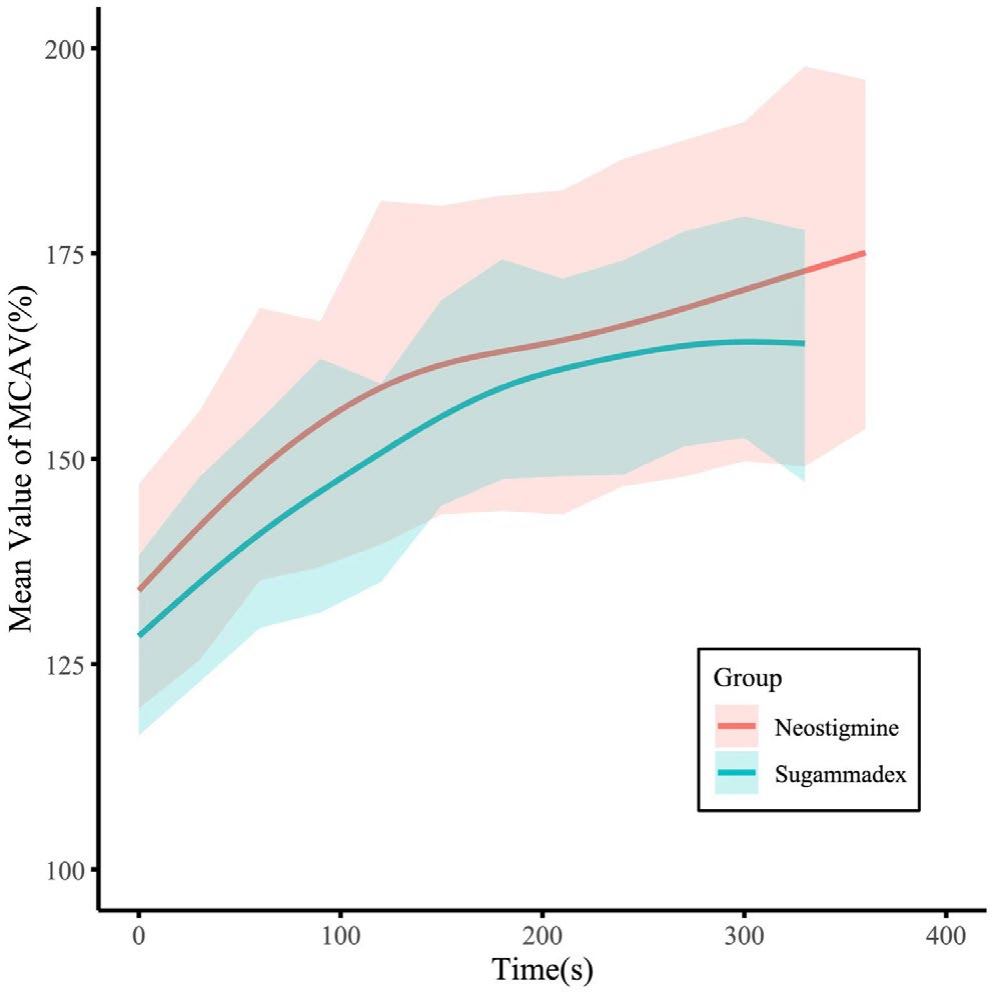
Line chart of mean MCAV% over time in the two groups. Note: The two curves are misaligned on the right horizontal axis because continuous trend curves were used, with the rightmost point representing the longest extubation time in each group.

The primary and secondary outcomes were presented in Table 2 (The details of outcomes were presented in the Table S2). For the primary outcomes, MCAV% S (24,600 [14,940–38,040] vs. 31,575 [24,210–50,040], *p* = 0.044) in the sugammadex group was significantly lower than that in the neostigmine group. We made a line chart showing the mean values of the two drugs over time (Figure 3), and it can be observed that the MCAV% S in the sugammadex group was lower than that in the neostigmine group across all time periods. After adjusting for sex, degree of stenosis on the operative side, collateral of the operative side, anesthesia time, MAP S, HR S, vascular clamp time, and the total volume of intraoperative infusions, the MCAV% area in the sugammadex group was persistently lower than that in the neostigmine group (estimate: −10,896; 95% CI: −21,387 ~ −405; *p* = 0.042). For secondary outcomes, there were significantly shorter eye‐opening time (51 [30–112] vs. 154 [59–354], *p* < 0.001) and extubation time (110 [75–150] vs. 237 [145–418], *p* < 0.001) of the sugammadex group than the neostigmine group. Additionally, no significant difference was observed in PetCO_2_ levels during the peri‐extubation period (39 [37–39] vs. 39 [37–40], *p* = 0.832) and five minutes after extubation (39 [38–40] vs. 39 [39–40], *p* = 0.144). Regarding postoperative 48‐h pulmonary complications, the rate of hypoxemia was significantly higher in the neostigmine group than the sugammadex group (12.2% vs. 31.7%, *p* = 0.033) (for details, see Table S2).

**TABLE 2:** Primary and secondary outcomes of CEA patients.

	Sugammadex (*n* = 41)	Neostigmine (*n* = 41)	*p*
Primary outcome			
MCAV% S (s・%), median (IQR)	24,600 (14,940, 38,040)	31,575 (24,210, 50,040)	0.044
Secondary outcomes			
Eye‐opening time (s), median (IQR)	51 (30, 112)	154 (59, 354)	< 0.001
Time to extubation (s), median (IQR)	110 (75, 150)	237 (145, 418)	< 0.001
PACU length of stay (min), median (IQR)	42 (35, 50)	38 (35, 50)	0.827
PetCO_2_ during peri‐extubation period, median (IQR)			
When extubation	39 (37, 39)	39 (37, 40)	0.832
5 min after extubation	39 (38, 40)	39 (39, 40)	0.144
Postoperative 48‐h CH, *n* (%)	3 (7.30)	8 (19.50)	0.105
Postoperative new cerebral infarction in MRI, *n* (%)	4 (9.80)	8 (19.50)	0.211
Postoperative 30‐day mRS Score, *n* (%)			0.109
0–2	29 (70.80)	35 (85.40)	
≥ 3	12 (29.30)	6 (14.60)	
30‐day any Stroke, Myocardial infarction or Death, *n* (%)	1 (2.40)	1 (2.40)	1

Abbreviations: CH, cerebral hyperperfusion; CI, confidence interval; DBP, diastolic blood pressure; HR, heart rate; IQR, inter‐quartile range; MAP, mean arterial pressure; PACU, post‐anesthesia care unit; PetCO2, end‐tidal carbon dioxide partial pressure; SBP, systolic blood pressure; TCD, transcranial Doppler.

### 3.2 Linear Regression Results

The results of the univariate linear regression are presented in Table 3, with those for all covariates included in the multivariate linear regression detailed in Table S4. In the univariate linear regression of MCAV% S, the sugammadex group showed a significant decrease in MCAV% S compared to the neostigmine group (estimate: −13,645; 95% CI: −25,208 to −2,082; *p* = 0.021). Extubation time showed a positive association with MCAV% S, with an estimate of 54.8 (95% CI: 24.5 to 85, *p* < 0.001). However, PetCO_2_ changes did not show a significant univariate relationship with MCAV% S (estimate: 48,315, 95% CI: −43,188 to 139,818, *p* = 0.3). Normality tests were performed on the residuals of the multivariate linear regression models prior to the analyses. A Kolmogorov–Smirnov test was conducted to evaluate the normality of model residuals, and the test results were D = 0.10 and *p* = 0.30, indicating that the residuals were approximately normally distributed. The Q‐Q plot and histogram of the residuals are presented in Figure S1 of the Supporting Information. After adjusting for sex, degree of stenosis on the operative side, collateral of the operative side, anesthesia time, MAP S, HR S, vascular clamp time, and the total volume of intraoperative infusions, the MCAV% area in the sugammadex group was persistently lower than that in the neostigmine group (estimate: −10,896; 95% CI: −21,387 ~ −405; *p* = 0.042). The positive association between extubation time and MCAV% S remained significant with an estimate of 55.12 (95% CI: 29.3 to 81.0, *p* < 0.001), while PetCO_2_ changes still had no significant relationship (estimate: 2,040, 95% CI: −80,679 to 11,094, *p* = 0.961).

**TABLE 3:** Linear regression analysis and mediation analysis of MCAV% S.

	Univariate analysis		Multivariate analysis^#^	
	Estimate (95% CI)	*p*	Estimate (95% CI)	*p*
Linear regression of MCAV% S				
Sugammadex vs. Neostigmine	−13,645 (−25,208~−2,082)	0.021	−10,896 (−21,387~−405)	0.042
Extubation time	54.8 (24.5~85)	< 0.001	55.12 (29.3~81.0)	< 0.001
PetCO_2_ changes	48,315 (−43,188~139,818)	0.30	2040 (−80,679~11,094)	0.961
Mediation of PetCO_2_ changes				
Average Causal Mediation Effects (ACME)	−60 (−2,781~1,732)	0.908	70.7 (−2,749~1,396)	0.900
Average Direct Effects (ADE)	−13,585 (−25,374 ~ −2,532)	0.014	−10,967 (−22,710~15.6)	0.052
Total Effect	−13,645 (−25,804~−2,689)	0.014	−10,896 (−22,521~−198.2)	0.046
Proportion Mediated	0.004 (−0.169~0.21)	0.914	−0.006 (−0.277~0.38)	0.918
Mediation of extubation time				
Average Causal Mediation Effects (ACME)	−6,709 (−13,244~−657)	0.03	−8,085 (−15,402~−2,075)	0.004
Average Direct Effects (ADE)	−6,936 (−20,386~4,813)	0.25	−2,811 (−15,434~8,052)	0.660
Total Effect	−13,645 (−24,921~−2,380)	0.02	−10,896 (−22,698~−1,260)	0.030
Proportion Mediated	0.492 (0.008~2.17)	0.05	0.742 (0.114~3.84)	0.034

^#^ Adjusted for sex, degree of stenosis on the operative side, collateral of the operative side, anesthesia time, MAP S, HR S, vascular clamp time, and the total volume of intraoperative infusions. ACME, Average Causal Mediation Effects; ADE, Average Direct Effects.

### 3.3 Mediation Analysis Results

The mediation of PetCO_2_ changes revealed that the average causal mediation effect (ACME) was not significant, with an estimate of −60 (95% CI: −2,781 to 1,732, *p* = 0.908) in the unadjusted model and 70.7 (95% CI: −2,749 to 1,396, *p* = 0.900) in the adjusted model. The average direct effect (ADE) was also not statistically significant (estimate: −10,967; 95% CI: −22,710 to 15.6, *p* = 0.052). However, the total effect remained statistically significant (estimate: −10,896; 95% CI: −22,521 to −198.2, *p* = 0.046). As for mediation of extubation time, the ACME was significant, with an estimate of −6,709 (95% CI: −13,244 to −657, *p* = 0.03) in the unadjusted model and –8,085 (95% CI: −15,402 to −2,075, *p* = 0.004) in the adjusted model. The proportion mediated was 0.492 (95% CI: 0.008 to 2.17, *p* = 0.05) in the unadjusted model and 0.742 (95% CI: 0.114 to 3.84, *p* = 0.034) in the adjusted model (Table 3).

### 3.4 ROC Analysis Results

**FIGURE 4:**
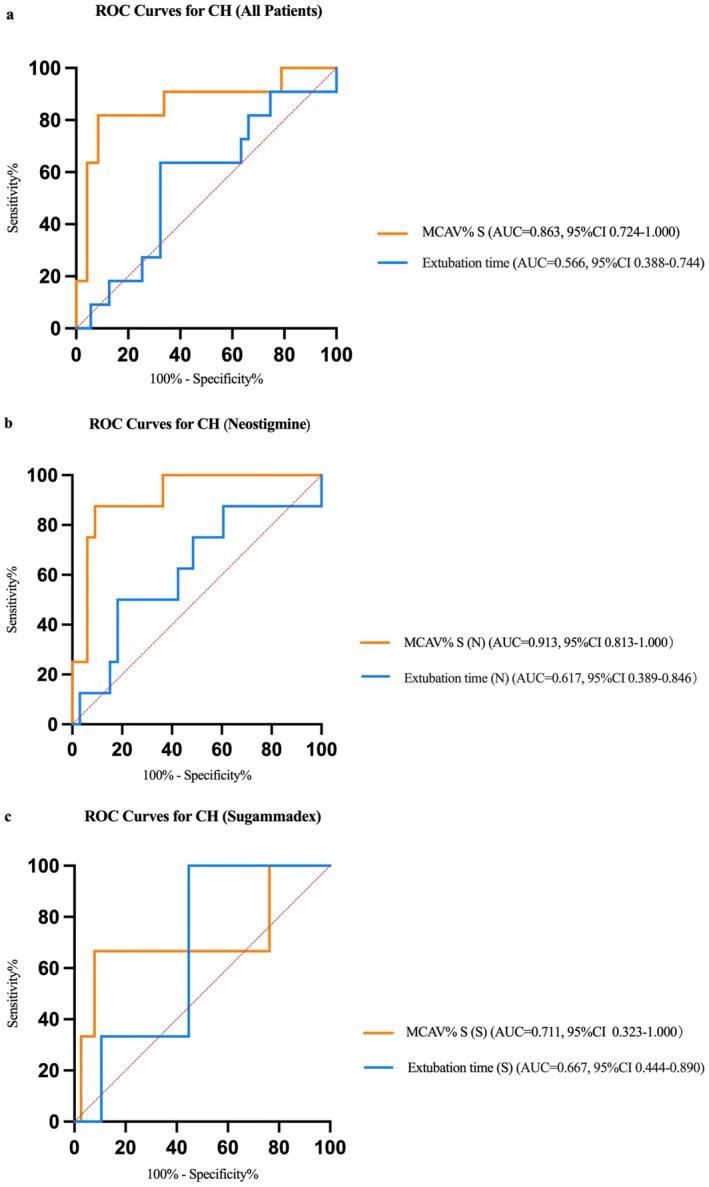
The ROC curves for postoperative 48‐h CH monitored by TCD in all patients (a), the neostigmine group (b), and the sugammadex group (c).

Furthermore, we conducted an exploratory analysis of the Receiver Operating Characteristic (ROC) curves for postoperative 48‐h cerebral hyperperfusion (CH) monitored by transcranial Doppler (TCD), as depicted in Figure 4. For all patients, the area under the curve (AUC) of mean cerebral artery velocity percentage (MCAV% S) was 0.863 (95% CI: 0.724–1.000, *p* < 0.001); the optimal threshold for differentiating between CH and non‐CH patients was 50257.5 s·%. The AUC of the time to extubation was 0.566 (95% CI: 0.3875–0.7444; *p* = 0.484). In the neostigmine group, the AUC of MCAV% S was 0.913 (95% CI: 0.8130–1.000, *p* < 0.001); the optimal threshold was 50257.5 s·%. The AUC of the time to extubation was 0.617 (95% CI: 0.3892–0.8456, *p* = 0.308). In the sugammadex group, the AUC of MCAV% S was 0.711 (95% CI: 0.3230–1.000, *p* = 0.230). The optimal threshold was 50,535 s·%. The AUC of the time to extubation was 0.667 (95% CI: 0.4436–0.8898, *p* = 0.342).

## 4 Discussion

In this single‐center, randomized controlled study, patients in the sugammadex group exhibited a shorter eye‐opening time and time to extubation, along with a lower incidence of postoperative hypoxemia when compared to those in the neostigmine group. To assess cerebral perfusion, we computed the MCAV% S. MCAV% S was defined as the area under the curve representing the percentage of the MCAV relative to the baseline during the peri‐extubation period, which reflects both the degree and persistence of cerebral hyperperfusion, enabling a more complete evaluation of the patient's overall exposure to potential risk during emergence from anesthesia. The findings indicated that the MCAV% S in the sugammadex group was lower than that in the neostigmine group. MCAV% S demonstrated favorable predictive performance for CH. Furthermore, the mediation analysis of MCAV% S revealed that the impacts of the two drugs on MCAV% S were primarily mediated through the extubation time.

During surgical anesthesia, it is essential for anesthesiologists to accurately determine the dosage of neuromuscular blocking agents and judiciously use reversal agents at the end of surgery, which not only ensures patient immobility during the procedure but also prevents postoperative CO_2_ accumulation. Sugammadex has been shown to reverse neuromuscular blockade more effectively and rapidly than neostigmine, leading to fewer postoperative complications, as confirmed by numerous studies [9, 10, 11, 12, 13]. Wu et al. [13], reported that sugammadex significantly reduced postoperative length of hospital stay and the incidence of postoperative chest imaging abnormalities in patients undergoing spinal surgery. Glenn et al. [11], found that in patients undergoing video‐assisted thoracoscopic surgery, the neostigmine group had significantly higher time to extubation, PACU length of stay, and percentages of patients with residual neuromuscular blockade (defined as TOF < 0.9) compared to those treated with sugammadex. Thomas et al. [14], conducted a prospective, randomized, double‐blind trial in high‐risk elderly patients, finding that a higher number of patients in the sugammadex group were diagnosed with imaging‐confirmed pneumonia. While there was a trend toward shorter hospital length of stay in the sugammadex group, it was only statistically significant in the Malaysian center. This study focused on patients with CEA, finding that sugammadex significantly reduced eye‐opening time and time to extubation, aligning with Glenn et al.'s findings. However, unlike the results from Wu and Thomas, sugammadex specifically reduced the incidence of postoperative hypoxemia, with no significant difference in the incidence of postoperative pneumonia.

CEA, as a neurosurgical procedure, demands absolute immobility during the operation. Given the specific requirements of vascular surgery, maintaining perioperative cerebral perfusion is crucial. Post‐operative cerebral hyperperfusion may cause severe problems such as cerebral hemorrhage and brain edema, posing significant risks to patients and even threatening life [4]. CH is closely associated with multiple factors. Existing literature shows that post‐operative cerebral hyperperfusion may be due to an incomplete circle of Willis, insufficient collateral circulation, reduced cerebrovascular reserve capacity, and suboptimal hypertension management [22, 23]. With the increasing use of intraoperative TCD monitoring, the importance of monitoring and managing intraoperative cerebral blood flow in preventing the occurrence of CH has become increasingly prominent [24, 25]. Intraoperative TCD monitoring can effectively indicate and predict the occurrence of CH after CEA [17, 26].

However, the entire anesthesia process also significantly influences cerebral blood flow. During anesthesia emergence, respiratory muscle strength gradually recovers. Poor recovery may result in inadequate ventilation, leading to CO_2_ accumulation and hypoxemia. Previous studies indicate that accumulation of CO_2_ elevates cerebral blood flow and causes dilation of the internal carotid and middle cerebral arteries [27], suggesting that elevated PaCO_2_ may impact cerebral perfusion during and after extubation. Therefore, muscle relaxant antagonists are vital in reducing CO_2_ accumulation during anesthesia emergence, shortening extubation time, thereby mitigating excessive cerebral blood perfusion and reducing the incidence of post‐operative CH. In this study, we used MCAV% S to represent the overall status of cerebral blood perfusion in patients during emergence. Our findings showed that the MCAV% S in the neostigmine group was higher than that in the sugammadex group. After multivariate regression analysis of variables such as gender, surgical‐side CAS, anesthesia duration, MAP, HR, and total intraoperative fluid infusion, a statistically significant difference in the MCAV% area between the two groups still existed. However, there was no significant difference in CO_2_ accumulation between the two groups. This suggests that sugammadex may be more effective than neostigmine in reducing cerebral perfusion during emergence in CEA patients, but the primary mechanism may not be through the reduction of CO_2_ accumulation during extubation.

The effects and underlying mechanisms of neuromuscular blockade and its associated agents on cerebral blood flow remain unclear. Previous investigations have indicated that neuromuscular blockade, regardless of whether atracurium or rocuronium is employed, does not induce clinically significant alterations in cerebral blood flow velocity, heart rate, or blood pressure [28, 29]. Nevertheless, these parameters may be influenced by the type of neuromuscular‐blocking drug and the depth of anesthesia [30, 31]. The impact of neuromuscular‐blocking antagonists on cerebral perfusion, particularly the central cholinergic regulation of cerebral blood flow, might be realized through the modulation of vasoconstriction and vasodilation [32, 33]. Although there is a paucity of research on the relationship between sugammadex and blood flow, it is postulated that this drug may have a relatively minor effect on hemodynamics [34]. Both drugs, however, can promote inspiration driven by respiratory muscle movement by antagonizing muscle relaxation. In our study, we compared these two drugs and did not observe a significant difference in their hemodynamic effects among patients undergoing CEA. Additionally, there was no notable disparity in CO_2_ responsiveness between the two drugs. Our mediation analysis of the MCAV% S further corroborated this finding. We discovered that the difference in MCAV% S was primarily attributable to the shortening of the extubation time rather than a reduction in the partial pressure of CO_2_. Moreover, it is undeniable that MCAV% S can effectively predict the occurrence of CH within 48 h after surgery. This suggests that MCAV% S could potentially serve as a detection indicator during the postoperative anesthesia recovery period to aid in the management of postoperative complications in patients.

Although CO_2_ is a potent cerebral vasodilator, our mediation analysis did not identify PetCO_2_ changes as a significant mediator of the effect on MCAV% S. This unexpected finding may be explained by several factors. First, PetCO_2_ was measured at only four time points, which may not capture dynamic CO_2_ fluctuations during emergence. Second, all patients received assisted ventilation during emergence, which may have attenuated intergroup differences in CO_2_ accumulation. Importantly, the significant mediation effect of extubation time suggests that shortening mechanical ventilation and the associated physiological stress during emergence may independently reduce cerebral hyperperfusion burden, beyond the effects of CO_2_ alone. Future studies using more precise peri‐extubation CO_2_ monitoring are warranted to further elucidate this relationship.

Beyond the mediation findings, the optimal MCAV% S threshold of approximately 50,257 s·% derived from our ROC analysis is based on a post hoc area‐under‐the‐curve calculation and therefore cannot be directly applied as a real‐time intraoperative alarm. This finding still holds several important clinical implications. First, it enables postoperative risk stratification: Patients exceeding this threshold during emergence are at higher risk for cerebral hyperperfusion (CH) within 48 h, warranting closer monitoring, stricter blood pressure control, and more frequent neurological assessments. Second, this threshold provides an objective, quantifiable endpoint for future interventional studies targeting cerebral hyperperfusion during emergence, facilitating standardized comparisons across studies. Third, while real‐time application of an AUC metric is not yet feasible, our findings establish a benchmark for developing real‐time surrogate indicators. Advances in continuous TCD monitoring and automated data acquisition may eventually allow real‐time calculation of cumulative exposure or time‐weighted average MCAV%. Future research could explore simplified metrics—such as duration above a baseline percentage or moving average of cumulative exposure—to trigger alerts when a patient approaches the risk threshold, helping translate this retrospective finding into a practical clinical tool. However, this hypothesis necessitates further verification in future studies.

With respect to vasoactive agents, although the dosage of ephedrine and phenylephrine differed between the two groups, these agents were typically administered during carotid clamping, and their hemodynamic effects had largely dissipated by the time of extubation. Therefore, we did not include the use of ephedrine and phenylephrine in the multivariate regression analysis.

There are several limitations to our study. Firstly, this study was conducted at a single center, which limits its generalizability. Secondly, it lacked a comparison group of patients who did not receive any reversal agents during the peri‐extubation period. In some cases, achieving immobility is possible with reduced neuromuscular blockade, and the TOF ratio may already meet extubation criteria at the end of the procedure. However, Liu et al. [35], observed that transcranial electrical motor evoked potential in spinal surgery patients was enhanced compared to those in patients without neuromuscular blockade and reversal agents. This suggests that the reversal of neuromuscular blockade by sugammadex could lead to more complete muscle strength recovery. Thirdly, due to equipment limitations, the sampling of IBP and HR during emergence was limited to once per minute, in contrast to the 30‐s intervals used for MCAV measurements, potentially reducing the accuracy of our results. Fourthly, the use of the ROC curve to predict postoperative 48‐h CH involved a small sample size and did not include patients who were not administered any reversal agents, which may compromise its broad applicability. Finally, this study did not include a formal cost‐effectiveness analysis. Given the significant cost difference between sugammadex and neostigmine, future research incorporating economic evaluations is needed to inform clinical adoption in various healthcare systems.

## 5 Conclusions

Our findings indicate that, in patients undergoing CEA, using sugammadex to reverse neuromuscular blockade during emergence is more advantageous than using neostigmine. Sugammadex can shorten the time to extubation, thereby reducing the excessive elevation of cerebral blood flow and potentially lowering the incidence of postoperative complications.

## Funding

Funding was provided by the National Natural Science Foundation of China (Grant No. 62376168), the National Natural Science Foundation of China (Grant No. 51775020).

## Ethics Statement

This study was approved by the Ethics Committee of Xuanwu Hospital, Capital Medical University (Approval No. [2023]140) on November 15, 2023. Written informed consent was obtained from all participants. The study was conducted in accordance with the Declaration of Helsinki.

## Conflicts of Interest

The authors declare no conflicts of interest.

## Supporting information


**Data S1:** Supporting Information.

**Data S2:** Supporting Information.

**Table S1:** Supplementary details for participant.
**Table S2:** Supplementary details for outcomes.
**Table S3:** Kolmogorov–Smirnov test.
**Table S4:** Regression analysis.
**Figure S1:** Residual Q‐Q plot and histogram of the multivariate linear regression model.

## Data Availability

Research data are not shared.
