# Supplementary figures and images for: Sugammadex Vs. Neostigmine for Cerebral Perfusion During Emergence From General Anesthesia in Patients Undergoing Carotid Endarterectomy: A Double‐Blind Randomized Controlled Trial
Source: CNS Neurosci Ther. 2026 May 18;32(5):e70924. doi: 10.1002/cns.70924 (PMC13181271; doi:10.1002/cns.70924)

## CONSORT 2010 Flow Diagram

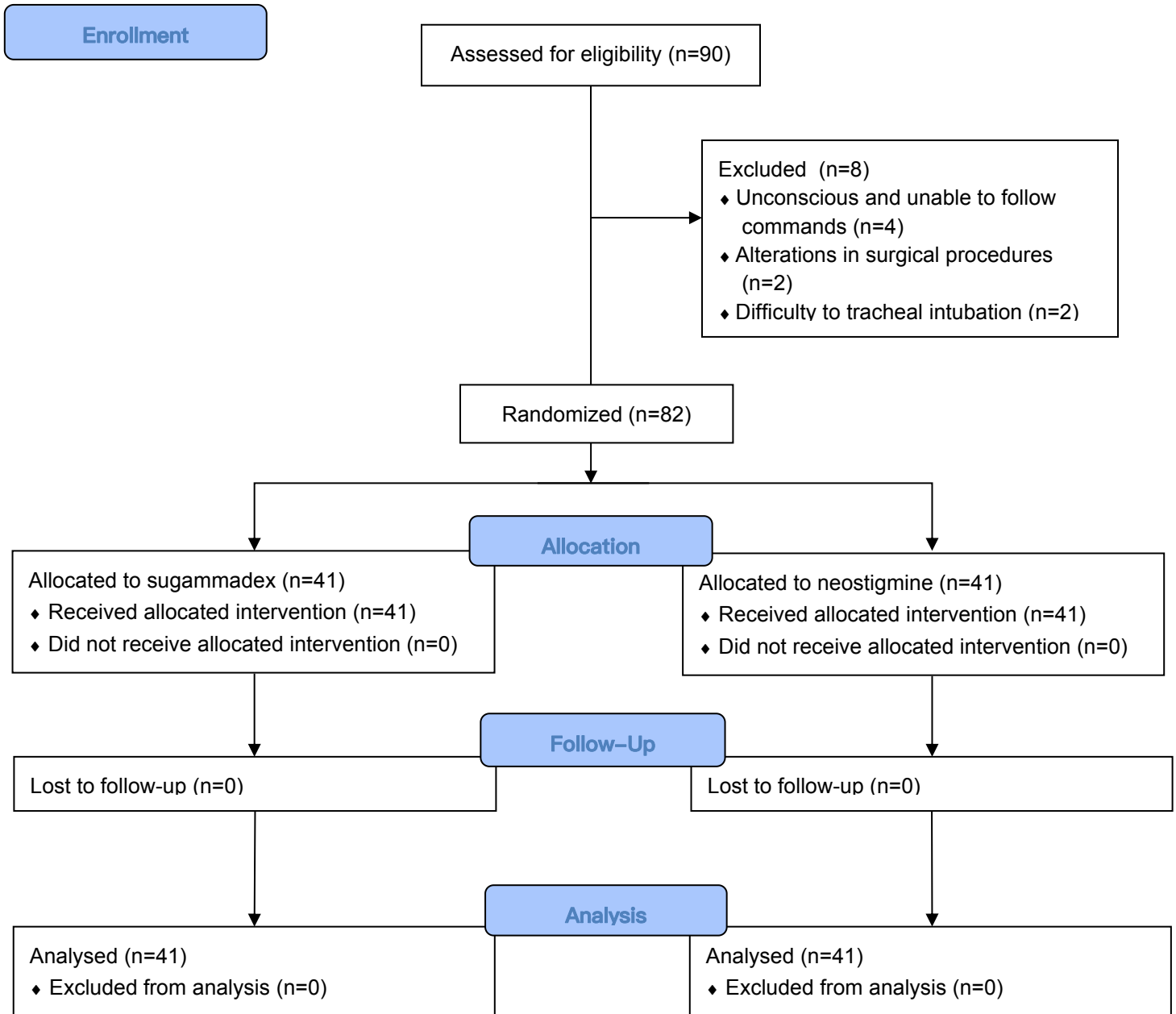

Figure 1 Consolidated Standards of Reporting Trials flow diagram.

Supplement: Supplementary file 2 — Data S2: Supporting Information. [file CNS-32-e70924-s001.pdf]
