# Supplementary material for: Sugammadex Vs. Neostigmine for Cerebral Perfusion During Emergence From General Anesthesia in Patients Undergoing Carotid Endarterectomy: A Double‐Blind Randomized Controlled Trial
Source: CNS Neurosci Ther. 2026 May 18;32(5):e70924. doi: 10.1002/cns.70924 (PMC13181271; doi:10.1002/cns.70924)

**Sugammadex versus Neostigmine for Cerebral Perfusion During Emergence from General Anesthesia in Patients Undergoing Carotid Endarterectomy: A Double-blind Randomized Controlled Trial**

Jia et al.

SUPPLEMENTARY DATA

**Catalogue**

**[General anesthesia protocol: 2](#_Toc1663)**

**[The detailed process of randomization and blinding: 4](#_Toc21737)**

**[Details of Exclusion criteria: 5](#_Toc25068)**

**[Details of Secondary outcomes: 6](#_Toc4364)**

**[Table S1 Supplementary details for participant characteristics. 7](#_Toc1127)**

**[Table S2 Supplementary details for outcomes. 9](#_Toc24212)**

**[Table S3 Kolmogorov-Smirnov test 12](#_Toc20478)**

**[Table S4 Regression analysis 14](#_Toc26077)**

**[Figure S1 Residual Q-Q plot and histogram of the multivariate linear regression model 15](#_Toc26656)**

# General anesthesia protocol:

Upon admission, patients underwent monitoring that included electrocardiography, pulse oximetry, non-invasive and invasive blood pressure (IBP), and BIS monitoring. Anesthesia was induced using propofol, sufentanil, and rocuronium, monitored by a Philips neuromuscular blockade monitor (865383 Philips, Shanghai, China). Before anesthesia induction, the patient’s forearm was secured, and the skin was degreased. Two surface electrodes were positioned on the ulnar nerve; the negative electrode near the wrist and the positive electrode 2~3 cm centripetally. A palm adapter connected the sensor and thermometer to the patient's palm, which remained immobile during monitoring. Endotracheal intubation was performed once neuromuscular blockade was confirmed with train-of-four count (TOFc) ≤ 1. After induction and stabilization of vital signs to baseline levels, ultrasound physicians calibrated the mean velocity of the middle cerebral artery (MCAV) using an EMS-9 PB ultrasound monitor (Delica, Shenzhen, China; software version V1.0, 1.6 MHz probe, 1.5 cm diameter, insonation depth of 40-65 mm, sample volume 6-10 mm), while anesthesiologists calibrated IBP and heart rate (HR). Maintenance of anesthesia involved propofol, remifentanil, and dexmetomidine, with norepinephrine used to maintain blood pressure within 100% to 120% of baseline levels. Rocuronium was administered in a dose of 0.2 mg/kg when the train-of-four ratio (TOFr) ≥ 0.2 and was not administered if less than 30 minutes remained in the surgery. The mean velocity percentage of the middle cerebral artery relative to the baseline (MCAV%) was maintained at no less than 50% of baseline values. After opening the diseased artery, adjustments were made to keep the MCAV% no higher than 200% of baseline. Throughout the operation, BIS levels were maintained between 40 and 60, and PaCO_2_ between 35 and 45 mmHg. At the end of the operation, the anesthetic was discontinued, and once TOFc reached ≥ 2, the appropriate reversal agents was administered (sugammadex 2 mg/kg for the experimental group, neostigmine 0.02 mg/kg combined with atropine 0.01 mg/kg for the control group). Extubation was considered when TOFr reached ≥ 0.9, indicating satisfactory recovery from neuromuscular blockade. MCAV was continuously monitored and recorded every 30 seconds during peri-extubation period. Patients were then transferred to the post-anesthesia care unit (PACU) for continued monitoring and oxygen therapy.During surgery, the TOF mode automatically measured neuromuscular activity at a frequency of 2 Hz, with a current intensity of 50 mA and a stimulation interval of 12 seconds.

# The detailed process of randomization and blinding:

The process of randomisation was performed by a research assistant who also followed the patient into the operating surgery to administer the study drug at the end of surgery, but who was not further concerned with the collection of postoperative data. The study drugs were prepared without this being witnessed by the attending anaesthetist and drawn up with normal saline to 10 ml in an unlabelled 10 ml syringe, and ensuring that the same volume of injection should be injected for the same patient regardless of which antagonist is used. Prior to extubation, an anesthesiologist, unaware of the grouping and not involved in drug preparation, administered an antagonist in the syringe according to the patient's weight. The collection of post-operative data was conducted by another experimentalist who was not aware of the study assignment.

# Details of Exclusion criteria:

In the exclusion criteria, "severe cardiac, pulmonary, hepatic, or renal dysfunction" is defined as follows:

**Severe cardiac dysfunction:** exclusion criteria using the New York Heart Association (NYHA) classification of cardiac function ≥ grade 3, or echocardiography showing the presence of severe mitral or tricuspid valve stenosis or regurgitation.

**Severe pulmonary dysfunction:** Lung Disease (GOLD) grades≥3, or the presence of poorly controlled bronchial asthma, active pulmonary inflammation were excluded.

**Severe hepatic dysfunction:** Patients with chronic liver disease and cirrhosis, specifically Child-Pugh Class B and C were excluded.

**Severe renal dysfunction:** Patients with chronic kidney disease (CKD) stage 3 or above or acute kidney injury (AKI) were excluded.

# Details of Secondary outcomes:

Eye opening time: time from administration of the reversal agents at surgery's end until eyes opening.

Time to extubation: time from the administration of the reversal agents until extubation.

Types of pulmonary complications within 48 hours post-operation are as followed. Each occurrence was assigned a score of 1 to facilitate the cumulative statistical score: (1) pneumonia, as defined by the United States Centers for Disease Control; (2) aspiration pneumonia, requiring both a detailed clinical history and radiological evidence; (3) atelectasis, identified via computed tomography or chest radiography; (4) pneumothorax, evidenced by clear clinical symptoms and radiological findings; (5) hypoxemia, characterized as oxygen desaturation with SpO2 ≤92% for >1 minute; (6) upper respiratory tract obstruction, confirmed by clear clinical symptoms and radiological evidence; (7) acute respiratory distress syndrome, as per the Berlin consensus definition.

| Table S1 Supplementary details for participant characteristics. |  |  |  |
| --- | --- | --- | --- |
|  | **Sugammadex (n=41)** | **Neostigmine (n=41)** | **p** |
| Operative side, n (%) |  |  | 0.331 |
| Left | 37 (90.20) | 34 (82.90) |  |
| Right | 4 (9.80) | 7 (17.10) |  |
| Smoking, n (%) | 35 (85.40) | 30 (73.20) | 0.173 |
| Drinking, n (%) | 32 (78.00) | 27 (65.90) | 0.219 |
| Cardiac disease, n (%) | 8 (19.50) | 11 (26.80) | 0.432 |
| Dyslipidemia, n (%) | 5 (12.20) | 8 (19.50) | 0.364 |
| Using hypertension drugs, n (%) | 29 (70.70) | 25 (61.00) | 0.352 |
| Calcium-antagonist drugs, n (%) | 20 (48.80) | 21 (51.20) | 0.825 |
| Beta-Blocker, n (%) | 1 (2.40) | 1 (2.40) | 1.000 |
| ACEI/ARB, n (%) | 16 (39.00) | 11 (26.80) | 0.240 |
| Diuretics, n (%) | 1 (2.40) | 0 (0.00) | 1.00 |
| Oral hypoglycemic agent, n (%) | 12 (29.30) | 11 (26.80) | 0.806 |
| Insulin, n (%) | 5 (12.20) | 6 (14.60) | 0.746 |
| Antiplatelet drugs, n (%) | 41 (100.00) | 41 (100.00) | 1.000 |
| Aspirin, n (%) | 41 (100.00) | 41 (100.00) | 1.000 |
| Clopidogrel, n (%) | 16 (39.00) | 17 (41.50) | 0.822 |
| Anticoagulant, n (%) | 1 (2.40) | 0 (0.00) | 1.000 |
| Warfarin, n (%) | 0 (0.00) | 0 (0.00) | 1.000 |
| Heparin, n (%) | 0 (0.00) | 0 (0.00) | 1.000 |
| Rivaroxaban, n (%) | 1 (2.40) | 0 (0.00) | 1.000 |
| Dabigatran, n (%) | 0 (0.00) | 0 (0.00) | 1.000 |
| Statins, n (%) | 41 (100.00) | 40 (97.60) | 1.000 |
| Preoperative laboratory testing |  |  |  |
| Hemoglobin（g/L）, median (IQR) | 140 (134, 148) | 142 (132, 150) | 0.770 |
| Erythrocyte count ×10^12^/L, mean±SD | 4.67 ± 0.45 | 4.56 ± 0.42 | 0.258 |
| White blood cell count ×10^9^/L, median (IQR) | 6.53 (5.70, 7.60) | 6.65 (5.68, 7.34) | 0.985 |
| Platelet count ×10^9^/L, mean±SD | 211.41 ± 47.48 | 226.95 ± 47.13 | 0.141 |
| Urea nitrogen (mmol/L), median (IQR) | 5.75 (4.74, 6.60) | 5.91 (5.19, 6.99) | 0.278 |
| Serum creatinine (μmol/L), mean±SD | 67.32 ± 14.43 | 65.05 ± 15.80 | 0.499 |
| SGOT (U/L), mean±SD | 23.49 ± 7.31 | 22.59 ± 5.76 | 0.537 |
| SGPT (U/L), median (IQR) | 27.00(20.00, 42.00) | 24.00 (19.00, 32.00) | 0.481 |
| FBG (mmol/L), median (IQR) | 5.46 (4.84, 8.29) | 5.87 (4.99, 6.82) | 0.588 |
| Na^+^ (mmol/L), median (IQR) | 142.00 (141.00, 143.00) | 141.00 (140.00, 143.00) | 0.062 |
| K^+^ (mmol/L), mean±SD | 4.15±0.36 | 4.11±0.30 | 0.544 |
| Cl^-^ (mmol/L), median (IQR) | 105.00 (104.00, 107.00) | 105.00 (103.00, 107.00) | 0.885 |
| Intraoperative dose of drugs |  |  |  |
| Propofol (induction) (mg), median (IQR) | 100.00 (75.00, 120.00) | 100.00 (100.00, 120.00) | 0.186 |
| Sufentanil (μg), median (IQR) | 20.00 (20.00, 22.50) | 20.00 (20.00, 21.00) | 0.176 |
| Propofol (maintenance) (mg), mean±SD | 56.77 ± 13.83 | 55.61 ± 17.17 | 0.738 |
| Remifentanil (μg), median (IQR) | 3056.00 (2544.00, 3592.00) | 3320.00 (2848.00, 3920.00) | 0.237 |
| Dexmedetomidine (μg), mean±SD | 36.24 ± 10.29 | 35.52 ± 11.06 | 0.760 |
| Intraoperative fluid (ml), median (IQR) |  |  |  |
| Crystalloid solution | 1200.00 (1100.00, 1300.00) | 1200.00 (1100.00, 1500.00) | 0.028 |
| Colloidal solution | 0.00 (0.00, 400.00) | 0.00 (0.00, 0.00) | 0.214 |
| Blood loss | 20.00 (20.00, 20.00) | 20.00 (20.00, 20.00) | 0.020 |
| Urine | 1000.00 (500.00, 1000.00) | 1000.00 (650.00, 1200.00) | 0.970 |

IQR, inter-quartile range; SD, standard deviation; ACEI, angiotension converting enzyme inhibitors; ARB, angiotensin receptor blocker; SGOT, serum glutamic oxaloacetic transaminase; SGPT, serum glutamic pyruvic transaminase; FBG, fasting blood glucose.

| Table S2 Supplementary details for outcomes. |  |  |  |
| --- | --- | --- | --- |
|  | **Sugammadex (n=41)** | **Neostigmine (n=41)** | **p** |
| The number of times MCAV% exceeded 200% during extubation | 0(0, 4) | 0(0, 5) | 0.772 |
| Whether MCAV% exceeds 200% ≥1 time during extubation | 19(46.30) | 18(43.90) | 0.824 |
| SBP S (s・mmHg), median (IQR) | 27(5, 65) | 52(16, 136) | 0.033 |
| DBP S (s・mmHg), median (IQR) | 5(0, 26) | 33(3, 49) | 0.017 |
| MAP S (s・mmHg), median (IQR) | 14(3, 37) | 42(5, 75) | 0.024 |
| HR S (s・bmp), mean±SD | 129±78 | 143±103 | 0.475 |
| PetCO2 during peri-extubation period, median (IQR) |  |  |  |
| When reversal agents were given | 35 (34, 36) | 36 (34, 37) | 0.104 |
| Opening eyes | 36 (35, 37) | 36 (35, 38) | 0.507 |
| Dose of intraoperative vasoactive agents |  |  |  |
| Noradrenaline (mg), median (IQR) | 0.69 (0.47, 1.16) | 0.78 (0.50, 1.21) | 0.562 |
| Ephedrine (mg), median (IQR) | 0.00 (0.00, 0.00) | 0.00 (0.00, 0.00) | <0.001 |
| Phenylephrine(μg), median (IQR) | 0.00 (0.00, 0.00) | 0.00 (0.00, 100.00) | 0.012 |
| Ebrantil(mg), median (IQR) | 15.00 (0.00, 25.00) | 10.00 (0.00, 25.00) | 0.697 |
| Nicardipine(mg), median (IQR) | 0.00 (0.00, 0.00) | 0.00 (0.00, 0.00) | 0.980 |
| Esmolol(mg), median (IQR) | 20.00 (0.00, 40.00) | 30.00 (0.00, 60.00) | 0.579 |
| Blood gas analysis, median (IQR) |  |  |  |
| pH |  |  |  |
| Upon admission, mean±SD | 7.41±0.02 | 7.42±0.02 | 0.840 |
| 5min after extubation, mean±SD | 7.34±0.06 | 7.33±0.04 | 0.328 |
| PACU for 30min, median (IQR) | 7.38(7.36, 7.40) | 7.38(7.36, 7.39) | 0.366 |
| PaCO_2_ |  |  |  |
| Upon admission, mean±SD | 39.98±2.84 | 39.37±3.03 | 0.352 |
| 5min after extubation, median (IQR) | 46.70(41.90, 52.50) | 47.40(44.40, 51.30) | 0.663 |
| PACU for 30min, mean±SD | 42.82±3.31 | 42.73±3.71 | 0.910 |
| PaO_2_ |  |  |  |
| Upon admission, mean±SD | 81.47±9.22 | 83.89±11.57 | 0.298 |
| 5min after extubation, median (IQR) | 107.00(83.80, 139.00) | 99.90(85.20, 122.00) | 0.469 |
| PACU for 30min, median (IQR) | 86.60(79.70, 98.90) | 86.10(75.70, 102.00) | 0.666 |
| Hemoglobin (g/L) |  |  |  |
| Upon admission, median (IQR) | 14.50 (13.90, 15.60) | 14.30 (13.70, 15.40) | 0.358 |
| 5min after extubation, median (IQR) | 13.80 (13.00, 14.90) | 14.00 (13.10, 14.60) | 0.974 |
| PACU for 30min, mean±SD | 13.79 ± 1.23 | 13.50 ± 1.49 | 0.344 |
| Na^+^ (mmol/L) |  |  |  |
| Upon admission, median (IQR) | 140.00 (140.00, 141.00) | 141.00 (139.00, 143.00) | 0.344 |
| 5min after extubation, median (IQR) | 142.00 (140.00, 144.00) | 143.00 (142.00, 145.00) | 0.036 |
| PACU for 30min, median (IQR) | 142.00 (139.00, 143.00) | 143.00 (141.00, 144.00) | 0.077 |
| K^+^ (mmol/L) |  |  |  |
| Upon admission, median (IQR) | 3.70 (3.60, 3.80) | 3.70 (3.60, 3.90) | 0.561 |
| 5min after extubation, median (IQR) | 4.20 (4.00, 4.30) | 4.20 (4.00, 4.40) | 0.907 |
| PACU for 30min, median (IQR) | 4.00 (3.90, 4.20) | 4.00 (3.70, 4.30) | 0.651 |
| Cl^-^ (mmol/L) |  |  |  |
| Upon admission, median (IQR) | 106.00 (105.00, 108.00) | 108.00 (106.00, 109.00) | 0.029 |
| 5min after extubation, median (IQR) | 109.00 (108.00, 111.00) | 110.00 (108.00, 111.00) | 0.203 |
| PACU for 30min, median (IQR) | 109.00 (107.00, 110.00) | 110.00 (109.00, 111.00) | 0.026 |
| Lac (mmol/L) |  |  |  |
| Upon admission, median (IQR) | 1.10 (0.80, 1.40) | 1.00 (0.80, 1.20) | 0.167 |
| 5min after extubation, median (IQR) | 1.10 (0.90, 1.40) | 1.00 (0.90, 1.30) | 0.689 |
| PACU for 30min, median (IQR) | 1.10 (0.90, 1.40) | 1.10 (0.90, 1.40) | 0.790 |
| BE (mmol/L) |  |  |  |
| Upon admission, mean±SD | 1.05 ± 1.83 | 0.75 ± 2.06 | 0.488 |
| 5min after extubation, mean±SD | -0.48 ± 2.21 | -0.57 ± 2.23 | 0.866 |
| PACU for 30min, median (IQR) | 0.40 (-0.80, 1.70) | -0.10 (-1.30, 1.20) | 0.149 |
| Hct (%) |  |  |  |
| Upon admission, median (IQR) | 44.50 (42.50, 47.70) | 43.90 (42.10, 47.30) | 0.373 |
| 5min after extubation, mean±SD | 42.69 ± 4.00 | 42.85 ± 5.72 | 0.887 |
| PACU for 30min, mean±SD | 42.24 ± 3.77 | 41.28 ± 4.92 | 0.320 |
| Postoperative 48-hour pulmonary complications |  |  |  |
| pneumonia, n (%) | 4(9.80) | 6(14.60) | 0.500 |
| Aspiration pneumonia, n (%) | 0(0.00) | 0(0.00) | 1.000 |
| Atelectasis, n (%) | 1(2.40) | 0(0.00) | 1.000 |
| pneumothorax, n (%) | 0(0.00) | 0(0.00) | 1.000 |
| Hypoxemia, n (%) | 5(12.20) | 13(31.70) | 0.033 |
| Upper respiratory tract obstruction, n (%) | 0(0.00) | 0(0.00) | 1.000 |
| Acute respiratory insufficiency, n (%) | 0(0.00) | 0(0.00) | 1.000 |
| Postoperative 48-hour CH-related symptoms, n (%) |  |  |  |
| swirl | 1(2.40) | 2(4.90) | 1 |
| headache | 1(2.40) | 5(12.20) | 0.203 |
| Hemispheric cerebral hemorrhage on the operative side | 0(0.00) | 0(0.00) | 1 |
| Redness and swelling of the head and face | 0(0.00) | 0(0.00) | 1 |
| Nausea and vomiting | 2(4.90) | 1(2.40) | 1 |
| Eye symptoms | 1(2.40) | 0(0.00) | 1 |
| convulsion | 0(0.00) | 0(0.00) | 1 |
| Transient neurological dysfunction | 3(7.30) | 2(4.90) | 1 |
| TCD over 200% | 3(7.30) | 8(19.50) | 0.105 |
| Postoperative 48-hour mRS Score |  |  | 0.319 |
| 0–2 | 28(68.30) | 32(78.00) |  |
| ≥ 3 | 13(31.70) | 9(22.00) |  |
| All-cause 30-day mortality, n (%) | 0(0.00) | 0(0.00) | 1 |
| Length of hospital stay (d), median (IQR) | 6(5, 6) | 6(5, 8) | 0.112 |
| Length of postoperative hospital stay (d), median (IQR) | 3(3, 4) | 3(3, 4) | 0.369 |
| Cost of hospitalization (￥), median (IQR) | 26223 (24654, 28894) | 27070 (24997, 32172) | 0.351 |

IQR, inter-quartile range; SD, standard deviation; PetCO_2_, end-tidal carbon dioxide partial pressure; PACU, post anesthesia care unit; PaCO_2_, arterial partial pressure of carbon dioxide; PaO_2_, arterial partial pressure of oxygen; Lac, lactic acid; BE, base excess; Hct, hematocrit.

| Table S3 Kolmogorov-Smirnov test | | | | | | |
| --- | --- | --- | --- | --- | --- | --- |
|  | Sugammadex (n=41) | | | Sugammadex (n=41) | | |
|  | Z-statistic | DF | p | Z-statistic | DF | p |
| Age (yr) | 0.12 | 41 | 0.147 | 0.095 | 41 | 0.200* |
| Rocuronium dosage (mg) | 0.095 | 41 | 0.200* | 0.123 | 41 | 0.122 |
| Anaesthesia time (min) | 0.111 | 41 | 0.200* | 0.117 | 41 | 0.173 |
| Operation time (min) | 0.12 | 41 | 0.144 | 0.114 | 41 | 0.200* |
| Vascular clamp time (min) | 0.196 | 41 | <0.001 | 0.173 | 41 | 0.003 |
| Total volume of intraoperative infusions (ml) | 0.227 | 41 | <0.001 | 0.184 | 41 | 0.001 |
| TOFr calibration value | 0.073 | 41 | 0.200* | 0.16 | 41 | 0.01 |
| MCAV% S (s・%) | 0.111 | 41 | 0.200* | 0.2 | 41 | <0.001 |
| Eye opening time (s) | 0.187 | 41 | <0.001 | 0.162 | 41 | 0.008 |
| Time to extubation (s) | 0.258 | 41 | <0.001 | 0.174 | 41 | 0.003 |
| PACU length of stay (min) | 0.138 | 41 | 0.046 | 0.177 | 41 | 0.002 |
| The number of times MCAV% exceeded 200% during extubation | 0.277 | 41 | <0.001 | 0.306 | 41 | <0.001 |
| SBP S (s・mmHg) | 0.197 | 41 | <0.001 | 0.179 | 41 | 0.002 |
| DBP S (s・mmHg) | 0.329 | 41 | <0.001 | 0.133 | 41 | 0.066 |
| MAP S (s・mmHg) | 0.236 | 41 | <0.001 | 0.131 | 41 | 0.075 |
| HR S (s・bmp) | 0.124 | 41 | 0.116 | 0.116 | 41 | 0.181 |
| PetCO2 during peri-extubation period |  |  |  |  |  |  |
| When extubation | 0.207 | 41 | <0.001 | 0.188 | 41 | <0.001 |
| 5min after extubation | 0.168 | 41 | 0.005 | 0.187 | 41 | <0.001 |
| Blood gas analysis |  |  |  |  |  |  |
| pH |  |  |  |  |  |  |
| Upon admission | 0.124 | 41 | 0.112 | 0.109 | 41 | 0.200* |
| 5min after extubation | 0.105 | 41 | 0.200* | 0.07 | 41 | 0.200* |
| PACU for 30min | 0.115 | 41 | 0.198 | 0.145 | 41 | 0.029 |
| PaCO2 |  |  |  |  |  |  |
| Upon admission | 0.09 | 41 | 0.200* | 0.107 | 41 | 0.200* |
| 5min after extubation | 0.093 | 41 | 0.200* | 0.142 | 41 | 0.037 |
| PACU for 30min | 0.126 | 41 | 0.097 | 0.096 | 41 | 0.200* |
| PaO2 |  |  |  |  |  |  |
| Upon admission | 0.062 | 41 | 0.200* | 0.131 | 41 | 0.072 |
| 5min after extubation | 0.21 | 41 | <0.001 | 0.195 | 41 | <0.001 |
| PACU for 30min | 0.162 | 41 | 0.009 | 0.248 | 41 | <0.001 |
| Cost of hospitalization (￥) | 0.386 | 41 | <0.001 | 0.23 | 41 | <0.001 |
| Length of hospital stay (d) | 0.271 | 41 | <0.001 | 0.154 | 41 | 0.015 |
| Length of postoperative hospital stay (d) | 0.387 | 41 | <0.001 | 0.358 | 41 | <0.001 |

*. Lower bound of true significance

| Table S4 Regression analysis | | | | | | | | | | |
| --- | --- | --- | --- | --- | --- | --- | --- | --- | --- | --- |
|  |  | unstandardized coefficients |  | Standardization coefficient | t | p | 95.0%CI for B value | | Collinearity diagnosis | |
|  |  | B | standard error | Beta |  |  | lower limit | upper limit | Tolerance | VIF |
|  | constant | -7335.984 | 18351.001 |  | -0.4 | 0.69 | -43901.151 | 29229.184 |  |  |
|  | Sex | -23146.461 | 7940.169 | -0.294 | -2.915 | 0.005 | -38967.592 | -7325.33 | 0.901 | 1.109 |
|  | Degree of carotid artery stenosis on the operative side | 16665.64 | 6935.359 | 0.233 | 2.403 | 0.019 | 2846.635 | 30484.644 | 0.969 | 1.032 |
|  | Anaesthesia time | 224.801 | 93.784 | 0.27 | 2.397 | 0.019 | 37.932 | 411.669 | 0.719 | 1.392 |
|  | MAP S | -89.301 | 70.329 | -0.133 | -1.27 | 0.208 | -229.435 | 50.834 | 0.833 | 1.2 |
|  | HR S | 87.436 | 30.632 | 0.295 | 2.854 | 0.006 | 26.4 | 148.472 | 0.855 | 1.169 |
|  | Total volume of intraoperative infusions | -8.215 | 11.966 | -0.08 | -0.687 | 0.495 | -32.059 | 15.628 | 0.669 | 1.495 |

dependent variable: MCAV% S

# Figure S1 Residual Q-Q plot and histogram of the multivariate linear regression model


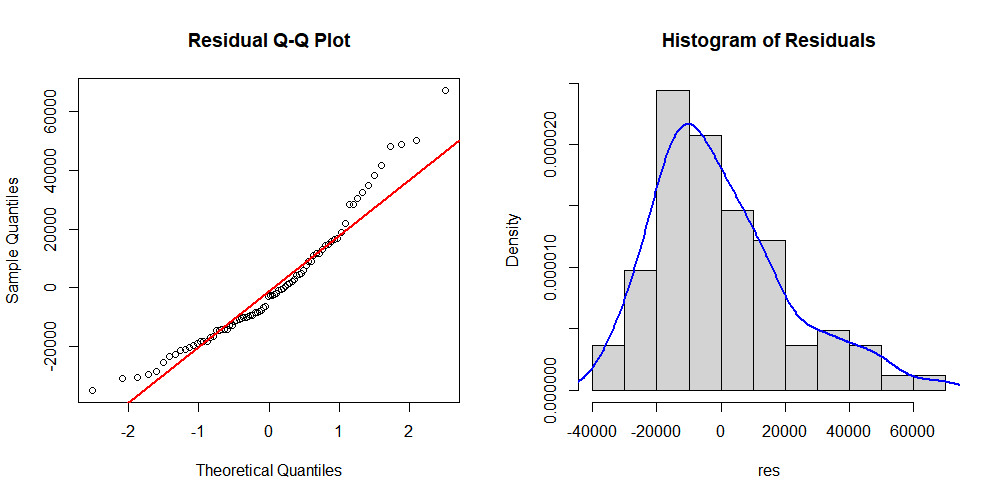

Supplement: Supplementary file 3 — Table S1: Supplementary details for participant. Table S2: Supplementary details for outcomes. Table S3: Kolmogorov–Smirnov test. Table S4: Regression analysis. Figure S1: Residual Q‐Q plot and histogram of the multivariate linear regression model. [file CNS-32-e70924-s002.docx]
